# How Do Phages Disrupt the Structure of *Enterococcus faecalis* Biofilm?

**DOI:** 10.3390/ijms242417260

**Published:** 2023-12-08

**Authors:** Magdalena Moryl, Antoni Różalski, Jose Antonio Poli de Figueiredo, Aleksandra Palatyńska-Ulatowska

**Affiliations:** 1 Department of Biology of Bacteria, Institute of Microbiology, Biotechnology and Immunology, Faculty of Biology and Environmental Protection, University of Lodz, 90-237 Lodz, Poland; antoni.rozalski@biol.uni.lodz.pl; 2 Department of Morphological Sciences, Federal University of Rio Grande do Sul—UFRGS, Porto Allegre 90010-150, Brazil; poli.figueiredo@outlook.com; 3 Department of Endodontics, Chair of Conservative Dentistry and Endodontics, Medical University of Lodz, 92-213 Lodz, Poland; aleksandra.palatynska-ulatowska@umed.lodz.pl

**Keywords:** biofilm, bacteriophages, *Enterococcus faecalis*, extracellular polymers

## Abstract

Biofilms are composed of multicellular communities of microbial cells and their self-secreted extracellular polymeric substances (EPS). The viruses named bacteriophages can infect and lyze bacterial cells, leading to efficient biofilm eradication. The aim of this study was to analyze how bacteriophages disrupt the biofilm structure by killing bacterial cells and/or by damaging extracellular polysaccharides, proteins, and DNA. The use of colorimetric and spectrofluorimetric methods and confocal laser scanning microscopy (CLSM) enabled a comprehensive assessment of phage activity against *E. faecalis* biofilms. The impact of the phages vB_Efa29212_2e and vB_Efa29212_3e was investigated. They were applied separately or in combination on 1-day and 7-day-old biofilms. Phages 2e effectively inhibited the growth of planktonic cells with a limited effect on the biofilm. They did not notably affect extracellular polysaccharides and proteins; however, they increased DNA levels. Phages 3e demonstrated a potent and dispersing impact on *E. faecalis* biofilms, despite being slightly less effective than bacteriophages 2e against planktonic cells. Phages 3e reduced the amount of extracellular polysaccharides and increased eDNA levels in both 1-day-old and 7-day-old biofilm cultures. Phage cocktails had a strong antimicrobial effect on both planktonic and biofilm-associated bacteria. A significant reduction in the levels of polysaccharides, proteins, and eDNA in 1-day-old biofilm samples was noted, which confirms that phages interfere with the structure of *E. faecalis* biofilm by killing bacterial cells and affecting extracellular polymer levels.

## 1. Introduction

*Enterococcus faecalis* constitutes a commensal microbiota in the oral cavity and gastrointestinal (GI) tract of humans and animals [1,2]. The bacteria possess the ability to persist in a broad range of environments, and they can act as a difficult-to-eradicate opportunistic pathogen in hosts. They cause bacteremia, meningitis, endocarditis, wound infections, and urinary tract infections (UTIs), including catheter-related ones [2,3,4]. They are also responsible for some dental infections, e.g., post-treatment apical periodontitis or refractory apical periodontitis [1,2]. *E. faecalis* causes many types of infections due to its ability to tolerate starvation and/or alkaline pH, form biofilms, and easily acquire antibiotic resistance [5].

Biofilms are formed by surface-attached or suspended aggregates of microbial, multi-species communities. They consist of bacterial cells, which constitute 10–25% of their total structure, but the main part is self-produced extracellular polymeric substances (EPSs). EPSs mainly include polysaccharides, proteins, eDNA, lipids, and other components in which bacterial cells are embedded [6]. Bacterial EPSs participate in the adhesion process, are necessary for biofilm matriculation, and stabilize its structure. Moreover, EPSs take part in the transport of nutrients to bacterial cells and in the regulation of biofilm dispersal processes. However, their most important function is to protect bacterial cells against harmful environmental influences [7]. The removal of biofilms from tissues or medical devices is extremely difficult because biofilm organisms are much less susceptible to environmental stresses [5,8,9] and are inaccessible to antibacterial agents, antiseptic irrigations, sanitizers, and the action of the host’s immune system [2,10]. Biofilms also provide a reservoir of bacterial cells in chronic infections [10]. The above-mentioned features are responsible for the mechanisms of adaptation and survival strategy of sessile bacterial cells. The bacterial cells in biofilm survive in the body for a very long time, and in favorable conditions (weak immune systems), they multiply, leading to reinfection. Nowadays, the implementation of antibiotics is the method of choice in the treatment of biofilm-related infections. Unfortunately, enterococci, especially *E. faecium* and *E. faecalis*, show a high level of resistance to antibiotics, including vancomycin (VRE), tetracycline, and erythromycin [2]. *E. faecalis* “conventional” antibiotic resistance, together with protective mechanisms that occur in biofilms, such as poor antibiotic penetration, starvation for a particular nutrient, slow cell growth, adaptive stress responses, and the formation of persister cells, confer multifactorial resistance and are responsible for the ineffectiveness of currently used therapies [3,11]. Thus, the high prevalence of multi-drug-resistant (MDR) bacteria requires further searching for suitable, alternative antimicrobial therapies [3]. Naturally virulent bacteriophages, the viruses that can infect and lyze the bacterial cells, can be used for the biocontrol of MDR biofilm-forming pathogens [5]. However, in phage therapy, temperate phages with a lysogenic cycle must be eliminated because they provide a resource for bacterial genetic diversity and bacterial evolution. Thus, lysogenic phages can contribute to an increase in bacterial antimicrobial resistance [12]. The advantages of phage therapy over antibiotics are already known. It is highly target-specific and non-toxic. Moreover, bacteriophages are characterized by a rapid multiplication rate, self-replicating capacity, and self-limiting nature. Phages can eradicate biofilms by disrupting the EPS matrix without perturbing the natural microbiota [3,13]. They encode a variety of enzymes, such as depolymerases and endolysins, which destroy the extracellular polymers protecting the structure of the cells [14,15]. Depolymerases are tail-spike proteins or free enzymes that specifically recognize polysaccharide residues. They bind and digest EPSs, which are mainly composed of polysaccharides. As a result, the activity of the enzymes leads to biofilm disruption and facilitates the penetration of virions deep into the biofilm structure by the bacterial cells. Subsequently, the phages kill bacteria in the lytic cycle. The cycle ends with the action of endolysins and peptidoglycan hydrolases, which cleave peptidoglycan and allow the release of bacteriophage progenies from the bacterial cells [15].

The application of bacteriophages faces various challenges, including a limited ability to target specific hosts–phages cannot target all pathogenic strains of a single bacterial species. Another problem is the occurrence of bacteriophage-resistant strains. The lack of pharmacokinetic data is also a problem in developing phage therapy. The preparations are difficult to standardize, and what is more, the effects of therapy are directly influenced by the mode of administration and dosage, creating challenges in the clinical application [16].

The objective of this study was to investigate the effect of the action of two bacteriophages, vB_Efa29212_2e and vB_Efa29212_3e, used separately and in combination, on different forms of *E. faecalis* bacteria. The ability of the phages to destroy planktonic cells and biofilms (1- and 7-day-old structures) with an emphasis on their action against particular components of the extracellular matrix of the biofilm (polysaccharides, proteins, and eDNA) was evaluated.

## 2. Results

### 2.1. Bacteriophages Action against E. faecalis Cells

#### 2.1.1. Planktonic Forms

**Figure 1:**
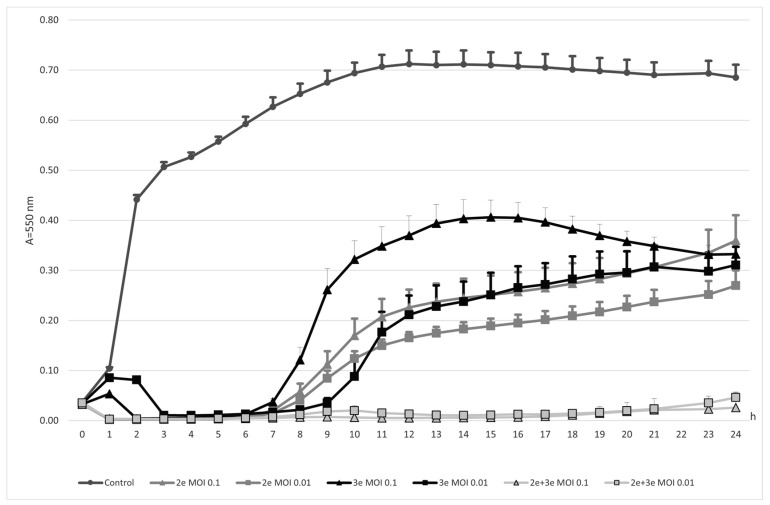
Bacteriophages action against *E. faecalis* planktonic forms.

Both investigated phages, vB_Efa29212_2e and vB_Efa29212_3e, alone or mixed together, had a strong inhibitory effect on *E. faecalis* (Figure 1). The strongest action was detected for the cocktail of the two studied phages (*p* ≤ 0.001). Bacteria were not proliferating for the whole period of the experiment (0–24 h). In the case of the phage cocktail, the multiplication of infection (MOI) value used in the experiment had no impact on the obtained results. Single phages also had lytic effects on *E. faecalis* cells. Phages 2e had a stronger effect compared to phages 3e in the 0–20 h time of the experiment. At MOI 0.01, no significant difference in the activity of phages 2e and 3e was observed. The inhibitory effect of monovalent phages weakened as the experiment continued (7–24 h). The use of phages at a lower titer (MOI 0.01) was more effective in combating planktonic forms of *E. faecalis.*

#### 2.1.2. Biofilm Decomposition

**Figure 2:**
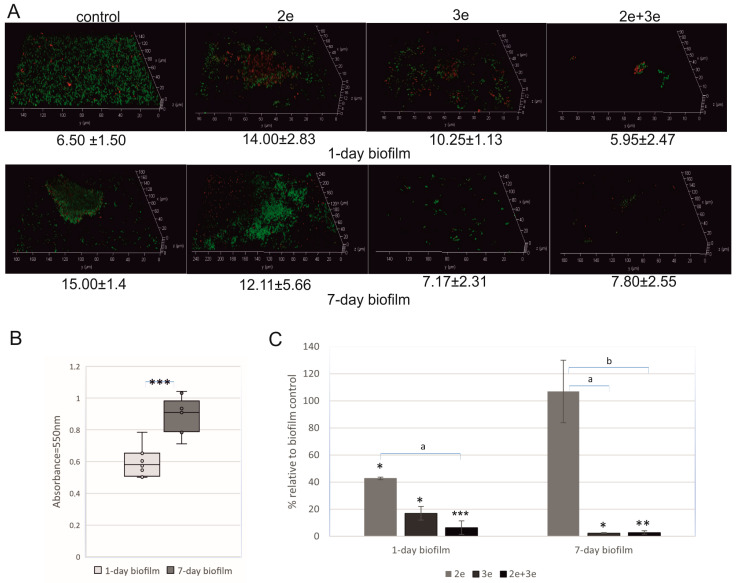
Bacteriophages’ action against *E. faecalis* biofilm; (**A**) Live–dead staining of biofilms; live cells (green) and dead cells (red); (**B**) the ability of *E. faecalis* to form biofilm (MTT test); (**C**) effect of phages on *E. faecalis* biofilm presented as percentages of reductions in the absorbance values in relation to control biofilms (100%); * values statistically significant at *p* ≤ 0.05 (versus control); ** values statistically significant at *p* ≤ 0.01 (versus control); *** values statistically significant at *p* ≤ 0.001 (versus control); a—values statistically significant at *p* ≤ 0.05; b—values statistically significant at *p* ≤ 0.01.

The studied *E. faecalis* strain had the ability to form biofilm on polystyrene plates. The biofilm biomass increased during the incubation, and after the 7th day of culture, it was significantly bigger than after the 1st day of incubation (*p* ≤ 0.001) (Figure 2A—control and Figure 2B). In the case of the 1-day-old biofilm, a thin layer of living cells (green color) was observed. In the 7-day-old biofilm, a typical structure of microcolony with a height of 15.00 ± 1.4 μm consisting of living cells was found (Figure 2A—control). The bacteriophage’s action against *E. faecalis* in 1- and 7-day biofilms is presented in Figure 2A,C.

The studied phages had a different action on sessile cells. Phage 2e destroyed cells in the 1-day biofilm; 60% lower absorbance (*p* ≤ 0.05) and dead (red) cells in the biofilm structure were observed (Figure 2A,C). Completely different results were obtained for the 7-day-old biofilm. No inhibitory effect of the phages on the biofilm cells was noted. Nevertheless, the structure of the biofilm was changed, e.g., high microcolonies were observed. Phages 3e considerably destroyed *E. faecalis* cells both in the 1- and 7-day-old biofilm structures (83% and 98% absorbance reduction, respectively, *p* ≤ 0.05). The best result was demonstrated for the phage cocktail; a 94% reduction in the 1-day biofilm (*p* ≤ 0.001) and a 97% reduction in the 7-day biofilm (*p* ≤ 0.01) were noted.

### 2.2. Bacteriophages Influence on EPSs in E. faecalis Biofilm

#### 2.2.1. Polysaccharides

**Figure 3:**
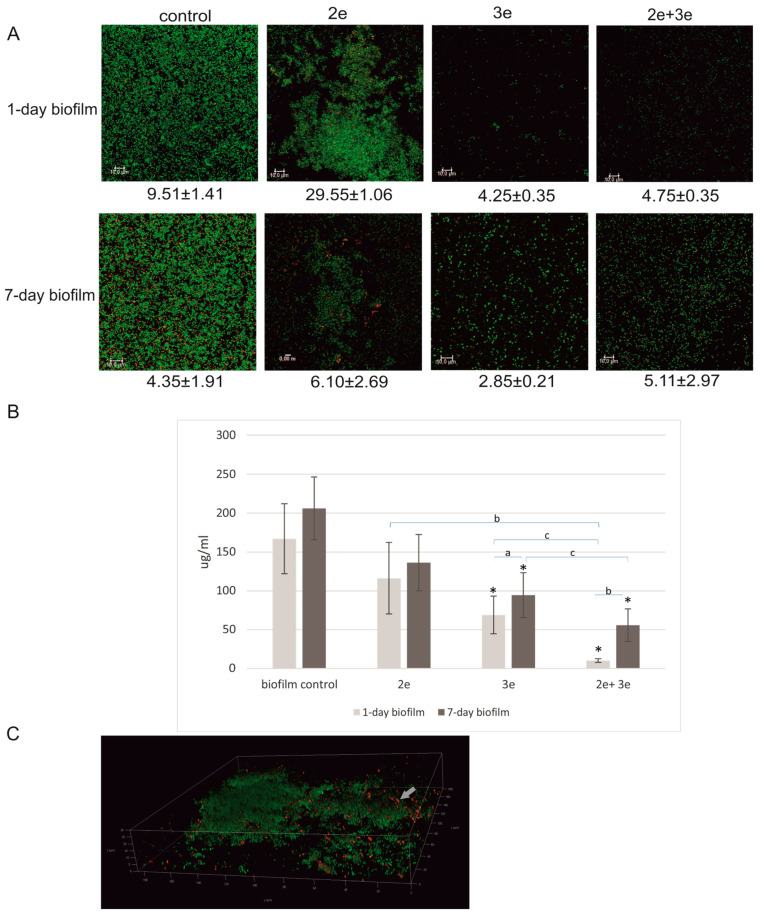
Bacteriophages’ influence on the level of polysaccharides in *E. faecalis* biofilm; (**A**) CLSM staining of biofilms treated with phages; bacterial cells (green) and polysaccharides fractions (red); (**B**) the level of polysaccharides in the biofilm treated with phages (phenol-sulphuryl method); (**C**) three-dimensional image of the biofilm with marked polysaccharides fractions (red colored fractions marked with a grey arrow); * values statistically significant at *p* ≤ 0.05 (versus control); a—values statistically significant at *p* ≤ 0.05; b—values statistically significant at *p* ≤ 0.01; c—values statistically significant at *p* ≤ 0.001.

The effect of the phage activity on the level of polysaccharides in EPS is presented in Figure 3A,C, showing the green-colored bacterial cells and red-colored polysaccharide fractions. The highest level of polysaccharides was recorded in the biofilm not subjected to the phage treatment (Figure 3B).

Phages 2e did not affect the polysaccharide level in the biofilm matrix; however, they influenced the structure of the biofilm. The biofilm did not cover the entire surface of the plate, but it formed high microcolonies containing fractions of polysaccharides, as shown in Figure 3A, especially in the case of the 7-day-old culture. Phages 3e and the cocktail of 2e and 3e had an inhibitory effect on the level of polysaccharides in the matrix (Figure 3B). In the case of the 1-day-old biofilm, the number of polysaccharides was reduced by 2.4 times and 17 times compared to the initial values, respectively (*p* ≤ 0.05), while in the 7-day-old biofilm, the drop was up to 2.2 times and 3.7 times, respectively (*p* ≤ 0.05). No significant differences were observed in the level of polysaccharides between the 1- and 7-day-old biofilms in the control conditions and after the treatment with 2e phages (Figure 3B).

#### 2.2.2. Proteins

**Figure 4:**
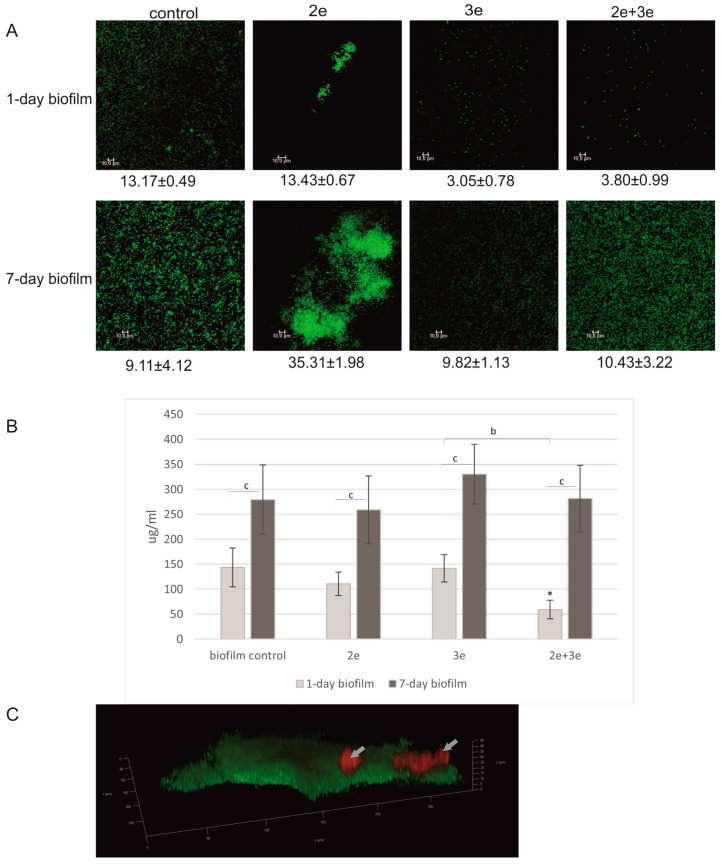
Bacteriophages’ influence on the level of extracellular proteins in biofilm; (**A**) CLSM staining of biofilms treated with phages; bacterial cells (green) and protein fractions (red); (**B**) the level of proteins in the biofilm treated with phages (Film Tracer SYPRO Ruby Biofilm Matrix Stain); (**C**) three-dimensional image of the biofilm with marked protein fractions (red colored fractions marked with grey arrows); * values statistically significant at *p* ≤ 0.05 (versus control); b—values statistically significant at *p* ≤ 0.01; c—values statistically significant at *p* ≤ 0.001.

The obtained results for the protein content in the EPS of *E. faecalis* biofilm after the tested phage implementation are presented in Figure 4.

Statistically significant differences in protein levels were observed between the 1- and 7-day-old biofilms without the phage application (*p* ≤ 0.001) (Figure 4B). Surprisingly, no significant differences in protein levels were observed in the biofilms treated with the phages. The exception was the 1-day-old biofilm treated with a phage cocktail, where a significant decrease (2.4 times) in the protein levels was noted when compared to the control (*p* ≤ 0.05). The biofilm structure with a red-marked extracellular protein fraction is presented in Figure 4A,C. Some changes in the biofilm structure occurred after the application of phage 2e. Distinct microcolonies of considerable height were visible. After the application of phage 3e and the phage cocktail, the structure was dispersed. Extracellular protein clusters were rarely observed (Figure 4C).

#### 2.2.3. eDNA

**Figure 5:**
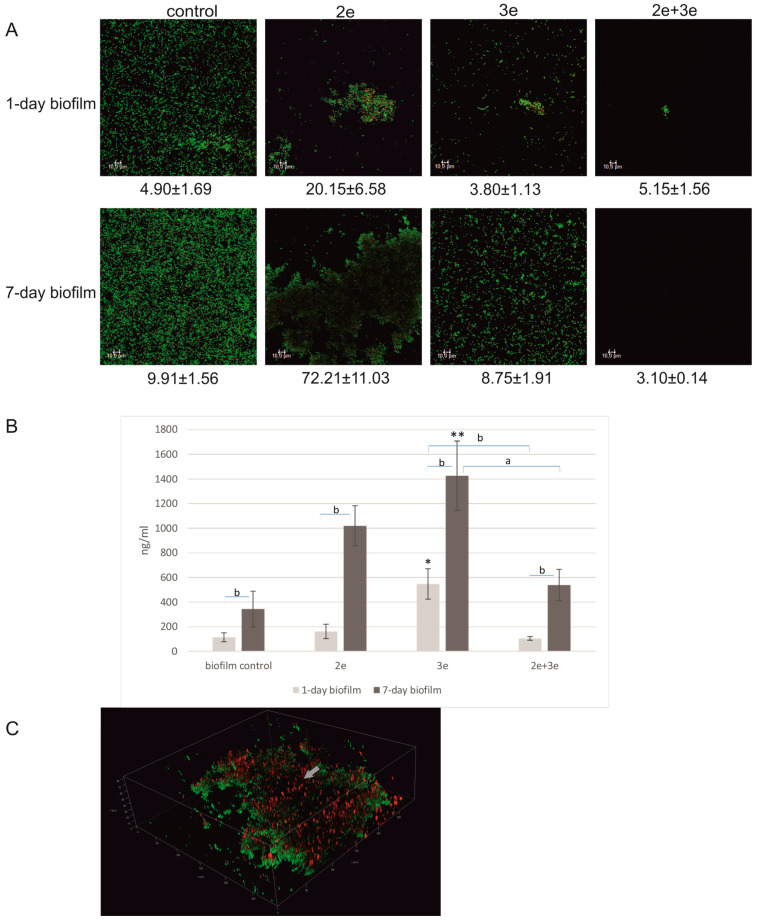
Bacteriophages’ influence on the level of eDNA in biofilm; (**A**) CLSM staining of biofilms treated with phages; bacterial cells (green) and eDNA fractions (red); (**B**) the level of eDNA in biofilm treated with phages (propidium iodide); (**C**) three-dimensional image of the biofilm with marked eDNA fractions (red colored fractions marked with grey arrows); * values statistically significant at *p* ≤ 0.05 (versus control); ** values statistically significant at *p* ≤ 0.01 (versus control); a—values statistically significant at *p* ≤ 0.05; b—values statistically significant at *p* ≤ 0.01.

The results for eDNA content after the tested phage application are presented in Figure 5.

The images presented in Figure 5A showed a biofilm structure with eDNA areas clearly marked in red. After the application of phage 2e, the structures were more compact and concentrated in microcolonies with clearly visible eDNA fractions. After the action of phage 3e and the phage cocktail, the biofilm structures were significantly dispersed. The eDNA levels in all the tested variants of the 7-day-old biofilms were significantly higher compared to the 1-day-old biofilm (*p* ≤ 0.01) (Figure 5B). The eDNA levels in both 1- and 7-day-old biofilms were significantly higher after the application of phage 3e. The greatest difference was found after the action of phage 3e on the 1-day-old biofilm, where the eDNA level was up to 4.8 times higher compared to the control (*p* ≤ 0.05). A slight decrease in the amount of eDNA (compared to the control) was observed only in one case—after the phage cocktail application on the 1-day biofilm. In the 3D image (Figure 5C), the location of eDNA (red) in the biofilm structure was shown.

## 3. Discussion

Bacteriophages are well-adapted to destroy bacteria, including biofilms, in their natural environment. First of all, phages can evolve, so they have the capacity for reciprocal adaptation to changes in their bacterial hosts through coevolution [17]. Secondly, they are able to replicate within their host, which leads to bacterial cell lysis and the release of more phages, gradually destroying the biofilm. Thirdly, they encode enzymes called depolymerases, which degrade components of the EPS matrix in biofilms. While infecting the biofilm, phages destabilize its EPS matrix, which allows them to penetrate and propagate within the biofilm. Some phages can also infect stationary-phase cells or even persister bacterial cells, remaining inside the cell until reactivation and then destroying them. Additionally, the enzymes encoded by phages, such as endolysins and holins, can break down bacterial cell walls, releasing viral particles and degrading bacteria and biofilm structures. Enzymes derived from phages can also target extracellular substances in encapsulated bacteria, helping in infection initiation and receptor binding. In summary, phages can disrupt preformed biofilms in several ways: by enzymatic degradation, cell lysis, and interfering with bacterial communication systems, such as quorum sensing. Understanding how phages interact with preformed biofilms is crucial for the development of phage-based therapies [18,19,20].

The aim of this study was to assess the effect of phage’s action on *E. faecalis* biofilm. In the presented work, two aspects were analyzed: how the phages interact with bacterial cells and how their activity influences the level of individual matrix components like extracellular polysaccharides, proteins, and DNA. In this study, the effects of the phages previously characterized by the authors, vB_Efa29212_2e and vB_Efa29212_3e, applied separately and in combination on 1-day and 7-day-old biofilms were evaluated [21]. Two variants of biofilms, which differed in their degree of maturity, were studied. The EPS matrix in a newly formed biofilm is less structured, and during maturation of the biofilm, it becomes less favorable to phage diffusion and penetration. It is the result of changes in the structure of the water channel network inside the biofilm [22]. The 1- and 7-day-old *E. faecalis* biofilms differed in thickness and structure organization. The longer biofilms grew, the higher levels of extracellular polymers (polysaccharides, proteins, and eDNA) were noted. Therefore, it was important to compare how the phages influence the biofilm structure at different stages of its maturity. Our study confirmed that phages had different effects on planktonic and biofilm forms of bacteria, and the maturity of the biofilm structure played a crucial role in the phage activity. We used two phages with different biological features [21]. Phages 2e were distinguished by a smaller virion size, a shorter latency period, and a higher burst size compared to phages 3e and demonstrated effectiveness against planktonic *E. faecalis* forms. The efficacy of phages 2e against biofilms was limited, particularly against mature (7-day-old) biofilm, where no activity was detected. Conversely, phages 3e, characterized by a longer latency period and a slightly lower burst size (compared to 2e), successfully eliminated both 1- and 7-day-old biofilms.

The results obtained for phages 2e are consistent with those found in many other studies, which indicate a reduced phage impact on biofilms when compared to planktonic forms [23,24,25]. There are many factors responsible for such results, which are associated with the spatial organization of biofilms, e.g., the presence of EPS. The decreased metabolic activity of bacterial cells within the biofilm may lead to a lower rate of phage replication [25]. Furthermore, deceased bacterial cells present within the matrix can serve as a nutrient source for surviving cells and offer them protection against phages. The phages can attach to receptors on these non-viable cells instead of the living ones [26,27].

On the other hand, the results obtained for phages 3e confirm those found in Goodarzi et al. studies (2022) describing the effectiveness of phages against mature *E. faecalis* biofilms [28]. The research conducted by Hanlon G.W. et al. (2001) also indicates that the sensitivity of *Pseudomonas aeruginosa* biofilm to phage F116 does not diminish with time or the age of the biofilm. This phage remained effective even against a 20-day-old biofilm [28,29].

The spectacular inhibitory effect of *E. faecalis* strains is achieved by a cocktail of phages that inhibits the proliferation of planktonic cells for 24 h and destroys 1- and 7-day-old biofilms of *E. faecalis*. The cocktails are used to increase a single phage lytic potential and to reduce the rate at which bacteria evolve phage resistance [26,30]. The exceptional effectiveness of the phage cocktail used in this study can be attributed to the application of two distinct phages against a single *E. faecalis* strain. These types of solutions serve as enhancements for the cocktail activity. The bacterial strain is incapable of mutation in order to develop cross-resistance to both phages [30]. Thus, the use of such a phage cocktail interferes with bacterial evolution and prevents the emergence of resistance genotypes that could evolve in the course of a single phage treatment. Many authors confirm the high effectiveness of phage cocktails against bacterial strains [31,32,33]. Chadha et al. (2016) suggest that the cocktail has great therapeutic potential in the treatment of multidrug-resistant infections of *Klebsiella pneumoniae* strains [31].

The research revealed that using a 0.01 multiplicity of infection (MOI) led to better phage propagation and resulted in a more effective bactericidal effect against planktonic forms of *E. faecalis.* The results obtained with phages of other bacterial species are diversified. Konopacki et al. (2022) indicate that a low initial MOI value has a significant impact on the high production of bacteriophages in *E. coli* strains. This phenomenon can be attributed to the greater abundance of host cells available for viral replication. When the MOI value remains high, the bacteria may be eliminated too rapidly, which prevents the achievement of a higher bacteriophage concentration [34]. However, this phenomenon does not always occur; e.g., in the study of Zhou et al. (2015), the application of *E. coli* phage JS09 at different MOI levels did not lead to any discernible differences in phage efficiency [35].

In the majority of biofilms, microbial cells make up 10 to 25% of the dry mass, with over 75 to 90% of the mass being attributed to the biofilm matrix [36]. The key components of EPS are polysaccharides and proteins, which are estimated to constitute approximately 40–95% of the matrix [37,38]. eDNA is also indicated as an omnipresent component in biofilms [39]. That is why a careful consideration of how phages act on biofilms is more than justified, including not only their impact on bacterial cells but also on EPS, which plays a significant role in biofilm development and stabilization. To date, there are not many data in the literature confirming the impact of phages on the levels of extracellular polymeric substances. EPSs form a critical scaffold, creating a three-dimensional matrix that provides protection and structural stability to bacterial cells, saving them from harsh environmental conditions. This biofilm matrix serves as a central hub, keeping all components together. It also facilitates cell-to-cell communication [36,40,41]. It has been reported that quorum sensing (QS) allows microorganisms to interact, regulates their density and behavior, and controls the biofilm formation process by creating extrapolymeric substances in biofilm [42]. Additionally, EPS contains extracellular enzymes that capture and use particulate matter from the surrounding environment as nutrients and energy sources. Due to cell lysis within the biofilm, the extracellular DNA release serves as a reservoir for horizontal gene transfer [36,40,41]. EPS is considered one of the most efficient natural defense mechanisms against phages. It can trap phage particles, making it harder for them to recognize bacterial receptors and initiate infection. The biofilm matrix also contains enzymes secreted by bacteria that can inactivate phages [43]. Taking all the above into account, the authors of the present study analyzed the influence of phage lysates on the levels of the basic EPS components. Tested levels of polysaccharides, proteins, and eDNA in biofilms rose with increasing culture time, and in the 7-day biofilm, the levels of EPS components (with the exception of polysaccharides) were significantly higher than in the 1-day biofilm. These findings are associated with the overall increase in biomass within the entire biofilm. As the biofilm matures, the extracellular matrix undergoes structural development [22].

The studied phages had various effects on the levels of polysaccharides in the EPS of *E. faecalis* biofilm. Phages 2e were found to encode polysaccharide depolymerases, but they did not exhibit a limiting effect on the level of these exopolymers in the 1- and 7-day-old biofilms. On the other hand, when phages 3e (which also encode polysaccharide depolymerases) or a phage cocktail were applied, significantly lower levels of polysaccharides were detected in biofilms. The reduction in the amount of these polymers was likely associated with a decrease in whole biofilm biomass due to the action of phage enzymes and their lytic activity. The degradation of biofilm polysaccharide elements following treatment with phages that encode depolymerases is known to result in diminished biofilm stability. This could lead to the release of bacteria from the biofilm or merely bring about a reduction in the biofilm’s composition without altering the count of viable bacteria [44].

To assess the protein levels in *E. faecalis* biofilm, the FilmTracer SYPRO Ruby Biofilm Matrix Stain reagent was used in the present work. It binds to glycoproteins, phosphoproteins, lipoproteins, fibrillary proteins, and calcium-binding proteins, which were the only ones detected in our experiment [45,46]. It was found that their levels in both 1-day-old and 7-day-old biofilms did not change (compared to control) after exposure to the tested phages. Despite a significant reduction in biofilm biomass following the action of phages 3e, the protein levels remained relatively stable.

Different results were obtained when assessing the level of eDNA in the *E. faecalis* biofilm. The phenomenon of phages releasing eDNA from bacterial cells has been described multiple times [27,39,47,48]. Also, in the presented study, larger amounts of this macromolecule were detected in biofilms subjected to phage treatment compared to the control, even in the presence of reduced biofilm biomass. Leroy et al. (2021) indicate that eDNA is not washed away but accumulated within the matrix, which supports the results of this trial [48]. It has also been demonstrated that a significant amount of eDNA in the matrix promotes the survival of cells in the biofilm, facilitating aggregation and bacterial cell adhesion. This exopolymer provides structural stability through numerous interactions with other EPS components. eDNA and polysaccharide intercellular adhesin PIA) can synergistically form a biofilm [39,47]. It is emphasized that the phage dosage must be appropriately selected in each therapy in order to avoid a real risk of promoting eDNA formation, leading to increased biofilm production [39].

All the above-listed unique properties make phages and phage-derived strategies promising for biofilm control. However, complete biofilm eradication with phages is challenging due to factors like impaired phage diffusion, biofilm cell metabolic activity, the presence of phage-resistant phenotypes, and limited phage host ranges that may hinder treatment success [20]. To conclude, the presented research highlighted and confirmed the significant role of phages, with the highest bactericidal potential of the phage cocktail in targeting both planktonic and biofilm forms of *E. faecalis*. The analysis of the results indicates that the efficacy of phages depends on the maturity of the biofilm and the biological features of bacteriophages. The phage lysates were found to affect both sessile bacterial cells and extracellular polymers of biofilm. The viruses promoted the accumulation of eDNA within the matrix while reducing the levels of polysaccharides. Nevertheless, further research is still needed to develop clear and effective protocols for this unique phage therapy for the eradication of *E. faecalis* biofilms.

## 4. Materials and Methods

### 4.1. Bacteriophages and Bacteria

An *Enterococcus faecalis* ATCC^®^ 29212TM strain was used in the studies. The bacteria were stored at −80 °C in brain heart infusion (BHI, Difco Laboratories, Detroit, MI, USA) and in 10% dimethyl sulfoxide (DMSO, Avantor, Gliwice, Poland) and then cultivated using BHI for 20 h at 37 °C.

Two virulent bacteriophages, vB_Efa29212_2e and vB_Efa29212_3e, were used in the study. The phages were isolated from urban wastewater from the Group Sewage Treatment Plant in Lodz, Poland. The filtered sewage samples were mixed with an equal volume of 2 × concentrated BHI and with *E. faecalis* culture. The sample was incubated at 37 °C for 24 h, then centrifuged (3600× *g* for 30 min) and filtered through a sterile filter (0.2 µm) [21]. The isolated phages were characterized. The morphology of phages and their biological features were determined, and the genome analysis was carried out [21]. Bacteriophage lysates with a titer of 10^9^ were stored at 5 °C.

### 4.2. Time Kills Assay—Bacteriophages Action against Planktonic Cells

The bacterial cultures and phage lysates were diluted in BHI to obtain 1 × 10^7^ CFU/mL and 1 × 10^6^ or 10^5^ PFU/mL, respectively. The MOI was 0.1 or 0.01. The agents in the wells of a 96-well, flat-bottomed polystyrene plate (Anicrin, Scorze, Italy) were mixed in equal volumes so that the final volume in the well was 200 μL. The absorbance was then measured on a plate reader at a wavelength of λ = 550 nm at time 0 and then every 1 h for 24 h. The time–kill curves were obtained.

### 4.3. Bacteriophages Action against Sessile Cells in Biofilm

#### 4.3.1. Biofilm Cultivation

*E. faecalis* was cultivated using BHI at 37 °C for 24 h. The culture was diluted in BHI to a density of 1 × 10^7^ CFU/mL, and 100 µL of each was added to the wells of a 96-well flat polystyrene plate. Next, the plate was incubated in a humid chamber at 37 °C for 24 or 168 h (one week). In the case of the long period of incubation, 70% of the substrate was replaced with fresh BHI every 48 h.

#### 4.3.2. Bacteriophage Application on Preformed Biofilm

After the incubation period, the biofilms formed on a 96-well flat polystyrene plate were washed with 120 µL sterile physiological saline to remove unbound bacterial cells. Bacteriophages were applied at a volume of 100 µL and a titer of 10^6^ PFU/mL. The phage cocktail was prepared by mixing equal volumes of both phages’ lysates of the same titer. The phage-untreated biofilm growth control of *E. faecalis* in BHI, a positive control (1% sodium hypochlorite, Avantor, Gliwice, Poland), and sterility controls were also prepared. Then, the plate was incubated in a humid chamber at 37 °C for 24 h.

#### 4.3.3. Biofilm Visualization

After the incubation period, the biofilms were rinsed with 120 µL of sterile saline, and the next 100 µL of BHI and 10 µL of 3-(4,5-dimethylthiazol-2-yl)-2,5-diphenyltetrazolium bromide (MTT) (Sigma-Aldrich, Saint Louis, MI, USA) reagent (concentration 5 mg/mL in PBS) were added [49,50]. The test involves the uptake and reduction of MTT by the oxidoreductase enzymes present in viable cells, resulting in the formation of formazan crystals. The plate was incubated at 37 °C for 30 min, after which the wells were emptied and 150 µL of DMSO (Avantor, Gliwice, Poland) and 25 µL of glycine buffer were added to dissolve the crystals. The absorbance was measured at 550 nm on a microtiter plate reader (Multiscan EX, Labsystems, Helsinki, Finland).

### 4.4. Estimation of Extracellular Polymers: Polysaccharides, Proteins, and eDNA in Biofilm

Biofilms were cultivated on polystyrene 96-well plates (transparent or black) in a manner analogous to that described above. The absorbance was measured on transparent plates or fluorescence on black plates (SPL Life Sciences, Pochon, Kyonggi-do, South Korea). The amount of total carbohydrate was determined using the phenol-sulphuryl acid method described by Dubois et al. (1956) with Masuko et al. (2005) modifications with glucose (Avantor, Gliwice, Poland) as a standard [51,52]. For this purpose, 50 µL of water was applied to the rinsed biofilm, and 150 µL of 96% sulfuric acid (Avantor, Gliwice, Poland) and 30 µL of 5% phenol (Avantor, Gliwice, Poland) were added, then mixed, heated at 90 °C for 5 min, and cooled on ice for 5 min. The absorbance was read at 492 nm using a microtiter plate reader (Multiskan EX Labsystems, Helsinki, Finland).

In order to assess the content of eDNA in the biofilm matrix, a standard curve of fluorescence versus DNA concentration was determined. The standard was lambda phage DNA (Sigma-Aldrich, Saint Louis, USA) with a starting concentration of 57.5 ng/μL, which was diluted in TE buffer (ThermoFisher Scientific, Waltham, MA, USA) to the appropriate concentrations from 5 to 1000 ng/mL and applied in a volume of 100 μL per well. To determine the content of extracellular DNA, propidium iodide (ThermoFisher Scientific, Waltham, MA, USA) at a concentration of 20 μM was used, which was added to the rinsed biofilm test samples so that the final concentration of the dye in the well was 6 μM. The plate was incubated for 15 min in the dark at room temperature, after which fluorescence was measured in a SpectraMax i3 (Syngen Biotech, Wroclaw, Poland) plate reader at an excitation wavelength of λ = 535 nm and an emission wavelength of λ = 617 nm [53,54].

The protein content (glycoproteins, phosphoproteins, lipoproteins, calcium-binding proteins, and fibrillary proteins) in biofilm was determined using the Film Tracer SYPRO Ruby Biofilm Matrix Stain (Invitrogen, Eugene, OR, USA) [45]. The standard was bovine serum albumin (Merck, Warsaw, Poland) with a starting concentration of 1 mg/mL in water. To determine the protein content, 10 μL of water and 100 μL of Sypro Ruby Red were added to the rinsed biofilm samples. The plate was incubated for 30 min in the dark at room temperature, and the fluorescence was measured in a SpectraMax i3 plate reader at excitation/emission wavelengths of 450/610 nm.

### 4.5. CLSM Visualization

*E. faecalis* biofilms were cultivated on a 24-well black plate with a glass bottom (Zell, Nörten-Hardenberg, Germany). A total of 500 μL of bacterial culture with a density of 1 × 10^7^ CFU/mL was added to each well and incubated at 37 °C for 24–168 h. Fresh BHI was supplied to the wells every 48 h. Next, the biofilms were washed with saline to remove planktonic bacteria, and bacteriophage lysates (monovalent or cocktail) with a titer of 10^6^ PFU/mL were added to the wells for 24 h. After the incubation at 37 °C, the biofilm was rinsed with distilled water and stained.

LIVE/DEAD^®^ BacLightTM Bacterial Viability Kit (Molecular Probes/Invitrogen, Eugene, OR, USA) was applied to study the effect of bacteriophages on sessile cells in biofilm structures because it enables differentiation of the bacterial population into live cells (green) and dead cells (red). The kit was used in accordance with the manufacturer’s instructions [55].

Double staining was applied to visualize bacterial cells and extracellular substances in the biofilm structure. SYTO 13 (Molecular Probes, Invitrogen, Eugene, OR, USA) at a concentration of 50 mM was used for 15 min to stain bacterial nucleic acids. Next, the biofilm was rinsed with distilled water, and WGA-TRITC lectin (Vector Laboratories, Newark, NJ, USA) at a concentration of 50 μg/mL was used for 30 min to stain extracellular polysaccharides (containing β (1→4)-N-acetyl-D-glucosamine residues) [7].

SYTO 13 at a concentration of 50 mM (15 min) and propidium iodide at a final concentration of 6 uM (20 min) were used for eDNA visualization. Furthermore, SYTO 13 and FilmTracer SYPRO Ruby Biofilm Matrix Stain were used for protein imaging according to the manufacturer’s instructions [53]. The plate was incubated for 30 min at room temperature. Next, the stained biofilms were rinsed to remove unbound dyes, and 100 µL of water was poured into the wells to prevent the cells from drying out. The imaging was performed using an SP8 confocal laser scanning microscope (Leica, Wetzlar, Germany) equipped with a 63× objective lens. The excitation/emission wavelengths were 488/514 nm for SYTO 13, 543/620 for WGA-TRITC, 535/617 nm for PI, and 450/610 nm for Sypro Ruby.

The image analysis was performed using the Leica Las-AF software (Version 3.3.0, Mannheim, Germany).

### 4.6. Statistical Analysis

Means and standard deviations (SD) were determined based on at least three independent repetitions of the experiments. Statistical analysis (Statistica 13.3, StatSoft Inc., Kraków, Poland) was carried out using the nonparametric one-way Anova test (Kruskal–Wallis). The test was used to determine if there were statistically significant differences between more than two independent groups. The nonparametric statistical test Mann–Whitney U was used to compare differences between two independent groups. The test was used for the data that were not normally distributed (as assessed by the Kolmogorov–Smirnov normality of the residuals test). Differences between the means of the two groups were considered significant at *p*-values ≤ 0.05 and highly statistically significant at *p*-values ≤ 0.01 and ≤0.001.

## Data Availability

Data are contained within the article.
